# Cardiac Emergencies in Neurosurgical Patients

**DOI:** 10.1155/2015/751320

**Published:** 2015-01-26

**Authors:** Tumul Chowdhury, Andrea Petropolis, Ronald B. Cappellani

**Affiliations:** Department of Anesthesiology and Perioperative Medicine, Health Sciences Center, University of Manitoba, Winnipeg, MB, Canada R3E 0Z2

## Abstract

Perioperative safety concerns are a major area of interest in recent years. Severe cardiac perturbation such as cardiac arrest is one of the most dreaded complications in the intraoperative period; however, little is known about the management of these events in the patients undergoing elective neurosurgery. This special group needs further attention, as it is often neither feasible nor appropriate to apply conventional advanced cardiac life support algorithms in patients undergoing neurosurgery. Factors such as neurosurgical procedure and positioning can also have a significant effect on the occurrence of cardiac arrest. Therefore, the aim of this paper is to describe the various causes and management of cardiac emergencies with special reference to cardiac arrest during elective neurosurgical procedures, including discussion of position-related factors and resuscitative considerations in these situations. This will help to formulate possible guidelines for management of such events.

## 1. Introduction

Perioperative safety is a major area of interest in recent years, with increasing emphasis on prevention, management, and outcome of cardiac emergencies. Cardiac arrest is a rare but dreaded intraoperative complication, with an incidence of 1.1 to 7.2 per 10,000 anesthetics [1, 2]. Intraoperative cardiac arrest can lead to devastating outcomes, and there has been renewed interest in its incidence, management, and outcomes [1, 2]. However, the management of intraoperative cardiac arrest during neurosurgical procedures remains poorly defined. This topic requires further attention, particularly because it is frequently neither feasible nor appropriate to apply conventional advanced cardiac life support algorithms (ACLS) during most of the neurosurgical arrests. Chest compressions and defibrillation may be challenging due to surgical positioning or exposure, and furthermore, appropriate management of cardiac arrest in neurosurgical patients may require immediate attention to the underlying cause rather than rote application of the ACLS algorithms. In addition, position and procedure have important effects on the occurrence as well as management of cardiac arrest in neurosurgical patients. Issues related to cardiac defibrillation and resuscitation in patients with skull pin fixation also require further attention.

Therefore, this paper is aimed at describing the various causes of cardiac arrest during elective neurosurgical procedures, as well as their management. It is also aimed at describing cardiac arrest in the context of neurosurgical positioning and resuscitative management in such situations. Special mention is given to resuscitative methods in patients with skull pin fixation. Finally, an algorithm for the management of cardiac arrest during elective neurosurgery is proposed.

## 2. Methods

### 2.1. Eligibility Criteria

Our aim was to identify any study describing management of intraoperative cardiac emergencies during elective neurosurgical procedures. Emergent, pediatric (<18 y), and obstetrical neurosurgical cases were excluded.

### 2.2. Definition

We have defined cardiac emergency as any event that includes ventricular fibrillation, pulseless electrical activity, asystole, and severe bradycardia (<40/min) with unstable blood pressure.

### 2.3. Literature Search

Studies were retrieved from Pub Med, Google, EMBASE, SCOPUS, and Web of Science, from 1 January 1970 to 31 May 2013. Searches were performed by two independent investigators (TC and AP), using combinations of the terms “cardiac arrest,” “cardiac asystole,” “ventricular fibrillation,” “pulseless electric activity,” “elective,” “intraoperative,” “cardiac resuscitation,” “neurosurgery,” and “defibrillation.” Searches were performed with no restriction on article type (original articles, reviews, retrospective studies, and case series/reports/letters) or article language. References were also checked for including potential papers related to the filed; however, duplicate results were deleted.

## 3. Causes and Mechanisms of Cardiac Emergencies

Intraoperative minor cardiac adverse events including bradycardia, hypotension, hypertension, and arrhythmias in neurosurgical patients are commonly reported and usually transient in nature; however, more serious cardiac complications including ventricular fibrillation (VF), asystole, and pulseless electrical activity (PEA) do occur in some patients and may produce catastrophic consequences if not managed promptly [7]. One must be cognizant of the causes and related mechanisms that incite these severe hemodynamic perturbations, as a good understanding of these variables forms the basis of appropriate management strategies. In this paper, we have categorized cardiac emergencies by the type of neurosurgical procedure.

### 3.1. Supratentorial Surgery

Severe hemodynamic disturbances are a frequently described phenomenon in patients undergoing elective supratentorial surgery. Most often, these disturbances occur as a result of various cranial nerve reflexes and were reported in both awake and anesthetized patients (Table 1). Among various causes, trigeminocardiac reflex (TCR) is the most common. TCR is a well-established brainstem reflex that can occur with stimulation of any sensory branch of trigeminal nerve and produces bradycardia, asystole, hypotension, apnoea, and gastric hypermotility. The afferent limb is formed by trigeminal nerve, whereas efferent fibers are carried by vagus nerve. Small internuncial nerve fibers of the reticular formation connect the afferent to the efferent premotor neurons located primarily in the nucleus ambiguus and the dorsal motor nucleus of the vagus. The reflex pathway activates cardioinhibitory parasympathetic vagal neurons, which terminate into the myocardium, which when activated cause negative chronotropic and inotropic responses [3, 4]. Other brainstem nuclei that are considered to be involved in TCR are parabrachial nucleus, the rostral ventrolateral medulla oblongata, the dorsal medullary reticular field, ventral superficial medullary dorsal horn (MDH), and the paratrigeminal nucleus. Any form of stimuli (mechanical, electrical, chemical, and/or thermal) can incite TCR. In general, stretch is the most powerful inciting factor and TCR events have been mainly reported during intense stimulation of the nerve directly or innervated structures; However, mild stimulation of trigeminal nerve (dural stimulation and skin traction) has been reported for inciting this reflex [3, 4]. Typically, these reflex-mediated disturbances resolve quickly with cessation of surgical stimulus, and advanced measures such as cardiopulmonary resuscitation are rarely required [3, 4]. If such an event occurs repeatedly during surgery, measures such as atropine administration or local anesthetic infiltration may be useful [3, 4]. However, refractory episodes may need intravenous adrenaline as well [3, 4].

Severe disturbances can also occur due to increased parasympathetic outflow related to surgical manipulation. Insular cortical stimulation, limbic stimulation, amygdala manipulation, and other brainstem stimulation have all been implicated in cardiac arrest during elective supratentorial surgery. Stimulation of amygdaloid complex may incite muscarinic receptors mediated cholinergic system and thus may produce these hemodynamic perturbations [5]. In particular, numerous cases of arrest during epilepsy surgery have been attributed to these causes [5, 6]. Again, most of the described cases resolve quickly with cessation of surgical stimulus, and treatment also frequently includes administration of atropine [5, 6].

Similarly, sudden increases in intracranial pressure (ICP) may also lead to severe hemodynamic disturbances via Cushing's reflex and may occur due to either anesthetic factors (coughing, gagging, light plane of anesthesia, high doses of volatile agents, hypercarbia, and hypoxia) or surgical factors (bleeding, seizures, and aneurysm rupture). Surprisingly, our literature search of elective supratentorial surgeries yielded a single case of asystole due to elevated ICP (hematoma) [7]. This case was successfully treated with immediate reopening of the dura and hematoma evacuation. On the other hand, as compared to raised ICP induced cardiac emergencies, intracranial hypotension has been described as a more frequent cause of severe cardiac perturbations in elective supratentorial surgical procedures including ventriculoperitoneal (VP) shunt insertion and extradural drain placement [8–11]. In these procedures either the rapid drainage of CSF or application of suction to the drains led to reverse herniation of brain and rapid release of suction reverted the cardiac changes in few cases; however, former case also required CPR [8–11].

Interestingly, irrigation with warm (42°C) and cold (20°C) saline solution has been implicated in sinus arrest and severe bradycardia, respectively, in elective supratentorial surgery, and termed as “temperature shock.” Amygdala and brainstem stimulation are the possible mechanisms of this rare phenomenon [5].

### 3.2. Skull Base Surgery

Among skull base surgeries (Table 2), cardiac emergencies were mainly reported in pituitary surgeries, microvascular decompression (MVD), and ablative procedures for trigeminal neuralgia [12–17, 19, 24, 57].

Asystole and severe bradycardia have been reported in transsphenoidal pituitary surgeries and are commonly attributed to the TCR [12–15, 17, 57]. The trigeminal nerve provides sensory innervation to both nasal tissues and the cavernous sinus, and TCR can occur with stimulation of any sensory branch of CN 5 along its pathway. This reflex mainly manifests as bradycardia, hypotension, asystole, and gastric hypermotility and may occur in up to 10−12% of transsphenoidal pituitary surgeries [15]. TCR has also been reported as a cause for severe adverse cardiac events in MVD for trigeminal neuralgia, occurring in 18% of patients in one study [24]. TCR has also been implicated in severe bradycardia, hypotension, and cardiac arrest occurring during ablation for trigeminal neuralgia [16]. However hemodynamic disturbances during ablative procedures may also be caused by severe vasovagal responses, and cardiac arrest has also been described during percutaneous thermocoagulation of the petrous ganglion of Andersch due to activation of the glossopharyngeal-vagal reflex (GVR) [19]. In summary, TCR-mediated cardiovascular events are common in skull base surgery and typically resolve promptly after the cessation of surgical stimulus. However, persistent or recurrent changes may require anticholinergic treatment and in some severe cases may require cardiopulmonary resuscitation [15, 16].

Cardiac arrest during skull base surgery has also occurred secondary to anterior hypothalamic stimulation. This has been described during surgical closure following transsphenoidal pituitary surgery, during which ongoing bleeding resulted in hypothalamic stimulation, increased parasympathetic outflow, and ultimately asystole requiring anticholinergic treatment and cardiopulmonary resuscitation [17].

### 3.3. Posterior Fossa Surgery

Many vital structures are contained in the limited space of posterior fossa, including the brainstem, floor of the fourth ventricle, and cranial nerves. There are multiple reports in the literature of reflex-mediated bradycardia and asystole related to surgical stimulation involving these structures (Table 3). A variety of intraoperative and postoperative cardiovascular complications can occur even with slight manipulations of the upper vagal rootlets and glossopharyngeal nerve. Most of the adverse cardiac events are reported in surgeries related to cerebellopontine tumors, Chiari malformation, cerebellar tumors, and the sitting position.

The majority of the events have been reported during tumor manipulation, and these are most often attributed to activation of neurogenic reflexes [21–23, 26, 27, 30]. TCR has been implicated in a large number of cases, and in one report, severe hemodynamic disturbances occurred in 11% of patients undergoing cerebellopontine angle (CPA) tumor resection [27]. There are also numerous reports of TCR-mediated arrest during CPA surgery, with asystole occurring secondary to tumor manipulation, cauterization of the cerebellar tentorium, trigeminal nerve manipulation, or trigeminal nerve traction [21–23]. Asystole can appear very abruptly, at times without antecedent bradycardia [23]. Hemodynamic changes due to TCR may be persistent, may progress to ventricular arrhythmia, and may in some cases be severe enough to necessitate cardiopulmonary resuscitation [21].

Glossopharyngeal nerve stimulation or glossopharyngeal vagal reflex (GVR) is also possible causes of cardiac arrest in posterior fossa surgery [26, 29]. In GVR, afferent signals are carried by the glossopharyngeal nerve while the vagus nerve carries efferent fibres, which stimulate the carotid sinus, leading to bradycardia, hypotension, and syncope [26, 29]. In patients who are severely symptomatic, it may be worthwhile to institute prophylactic measures such as application of topical or intravenous local anesthetic, as well as preemptive atropine administration; however asystole may still occur [29]. Prophylactic transvenous pacemaker placement has also been used in severely symptomatic patients, though this may not be necessary as cardiac rhythm usually returns to normal after brief periods of surgical interruption [29].

Similarly, stimulation of the vagus nerve itself may lead to severe hemodynamic disturbances including bradycardia, bundle branch block, and asystole [26, 30]. These events have been described during cerebromedullary schwannoma resection, as well as vagal rootlet section for glossopharyngeal neuralgia [26, 30]. These disturbances may occur despite pretreatment with atropine and, interestingly, abnormal cardiac rhythms (RBBB) may persist for days following vagal rootlet section [30]. Ventricular fibrillation arrest has also been attributed to vagal stimulation and resultant coronary vasospasm during CPA surgery [20]. This patient was found to have clear coronary arteries on postoperative angiogram [20].

Brainstem stimulation has also been implicated as a cause for asystole during posterior fossa decompression and has been successfully treated with cessation of surgical stimulus as in conjunction with atropine administration [25].

Venous air embolism (VAE) has also been reported as a cause of bradycardia and asystole in posterior fossa surgery performed in the prone position [28]. In this case, the most likely cause of the hemodynamic disturbances was felt to be paradoxical air embolism involving the coronary arteries [25].

### 3.4. Cerebrovascular Surgery

Cardiac perturbations including electrocardiographic abnormalities, hypertension, and arrhythmias are commonly described in aneurysmal subarachnoid hemorrhage [31–34, 58]. There are many reports of cardiac catastrophic events during aneurysm clipping or arteriovenous malformation surgery, and these are frequently attributed to centrally mediated sympathetic surges (Table 4).

Asystole during cerebrovascular surgery has been attributed to TCR in multiple cases [31, 32]. Transient asystole has occurred during anterior communicating artery aneurysm clipping, at the time of dural detachment from the sphenoid ridge [31]. This was attributed to TCR occurring secondary to stimulation of the middle meningeal branch of the trigeminal nerve. Similarly, TCR has been reported as a cause for asystole during clip placement on the supraclinoid portion of the internal carotid artery (ICA) as well [32].

Myocardial ischemia has also been described as a cause of cardiac arrest in this group. As in any kind of surgery, this can occur secondary to coronary artery disease and reflects the demand and supply disbalance or acute plaque rupture [34]. However, coronary vasospasm has also been described as a cause of severe hemodynamic disturbances associated with ST abnormalities during aneurysm clipping [33]. Such hemodynamic disturbances include bradycardia with complete atrioventricular blockade, ventricular tachycardia, pulseless electrical activity (PEA), and hypotension [33]. The vasospasm was attributed to increased vagal output with dural and cranial closure, although medications (propranolol, prostaglandin E1) may also have been contributing factors [33].

### 3.5. Spine Surgeries

Hemodynamic perturbations can be anticipated in spine surgeries due to various causes including prone positioning, bleeding, and spinal shock. However, in addition to bleeding, cardiovascular emergencies may be related to stimulation of specific nerve roots, autonomic dysreflexia or rarely due to venous air embolism and often require intensive resuscitative efforts including chest compressions (Table 5). The majority of these adverse events are reported in either cervical or upper thoracic spine surgeries.

There are multiple reports of severe hemodynamic disturbances occurring secondary to neurogenic causes during spine surgery [38, 40, 53]. This has occurred secondary to nerve root stimulation during repair of a C7-T1 disc herniation [38]. The proposed mechanism was diminished activity of the supraspinal sympathetic control system and hyperactivation of the parasympathetic system leading to asystole [38]. Cardiac arrest has also occurred during thoracoscopic sympathectomy, with arrhythmias including asystole and ventricular fibrillation occurring at the time of sympathetic trunk transection [40, 42].

There are also multiple reports of venous air embolism as a cause for cardiac arrest in elective spine surgery [39, 43, 45]. This has occurred in scoliosis surgery, lumbar laminectomy, and decompression for invasive spine tumor [39, 43, 45]. Resuscitation is often difficult in these cases, and outcomes frequently are poor [43, 45]. Multiorifice central venous catheters are sometimes used, but attempted aspiration of air from the right atrium may be unsuccessful, even in cases where right atrial air is present on postmortem examination [43]. Other types of embolic events have also been described during spine surgery, including fatal fat embolism during bone cement injection during percutaneous vertebroplasty [37].

Cardiovascular collapse during spine surgery can also occur due to vascular injury and acute blood loss, and multiple cases have been reported during lumbar spinal surgery [59]. The most commonly injured vessel is the left common iliac artery, which is situated immediately anterior to the fourth lumbar intervertebral disc [59]. However, injury may alternatively involve the right common iliac artery, iliac veins, aorta, or inferior vena cava [59]. Bleeding may be apparent at the surgical site but can also be occult, with bleeding into the abdomen or retroperitoneum [41, 59, 60]. Multiple fatalities due to serious vascular injury have been described in lumbar spine surgery [41, 44]. In one review, this type of injury resulted in death in seven out of 24 cases, despite aggressive resuscitation and laparotomy [41].

Cardiac arrest has also occurred during ethanol injection of symptomatic vertebral hemangioma [36]. This episode was attributed to intravascular ethanol injection, resulting in sinus node depression [36].

### 3.6. Drug-Induced Cardiac Arrest

There are multiple reports of drug-induced cardiac arrest in elective neurosurgical patients. These are frequently related to hemodynamic effects of various drugs that are commonly used in neurosurgery, including dexmedetomidine, remifentanil, and phenytoin (Table 6). Dexmedetomidine, a highly selective alpha-2 agonist, commonly produces negative cardiovascular changes including bradycardia and hypotension and has been linked to cardiac arrest in elective neurosurgery [47]. Remifentanil is another agent that has been identified as a possible contributor to severe hemodynamic disturbances [22]. However in reported cases of cardiac arrest occurring with these drugs, authors have been unable to rule out neurogenic causes such as elevated ICP, TCR, and brainstem compression [47]. Similarly, other medications such as propofol and metoprolol have been associated with cardiac arrest in elective neurosurgical cases [49]. Nonetheless, drugs producing negative cardiovascular changes necessitate the use of invasive hemodynamic monitoring and should be used cautiously in elderly patients and patients with preexisting cardiac disease.

Phenytoin, which is commonly used in neurosurgery for seizure prophylaxis, has also been implicated in serious cardiovascular events [51, 52]. This has been described in the setting of phenytoin overdose or rapid infusion of phenytoin, due to sodium channel blockade and effects on cardiac conduction and contractility [51, 52]. Cardiac arrest has also been described as a paradoxical response to ephedrine bolus in a patient receiving a phenytoin infusion [51]. This was attributed to potential interactions between phenytoin and ephedrine.

Locally applied papaverine, which has been used for the prevention of cerebral vasospasm, has also been implicated in fatal cardiac arrest [48]. This was thought to be due to stimulation of the hypothalamus or vagal nucleus, or with brainstem depression, although the exact mechanism was not known.

Anaphylaxis is also a possible cause for cardiac arrest in neurosurgery, as in any kind of surgery. This has been described following irrigation with saline bacitracin [61]. Cardiac arrest due to electrolyte abnormalities has also been described. This occurred in a patient taking losartan preoperatively, who also had a history of consuming large amounts of potassium-rich foods [50]. Such events could perhaps be avoided with preoperative screening of serum electrolytes.

## 4. Position-Related Factors

Neurosurgical positioning can present significant challenges during cardiovascular resuscitation. Procedures are frequently performed in positions other than supine, such as head up, semisitting, sitting, prone, and lateral (Table 7). In neurosurgery, cardiovascular disturbances are frequently reflex-mediated and rapidly resolving; however if chest compressions or defibrillation is required, position and surgical field related factors make resuscitation more difficult.

There are numerous reports of cardiac arrest requiring chest compressions in patients undergoing elective neurosurgery in the prone position [25, 35, 37, 39, 43, 44, 46, 50]. In most cases, patients were turned supine for chest compressions; however, this is not always immediately possible, due to skull pin fixation, halo fixation, open surgical wounds, or protruding hardware [25, 35, 37, 39, 43, 44, 46, 50]. Prone chest compressions have been successfully applied between the scapulae, and this maneuver can be used while preparations are being made to turn the patient supine. Effective compression can be given while placing fist or firm support behind the sternum; however, it can also be challenging while patient is on bolsters/Wilson's frame [2]. One can also anticipate further difficulty if patient is positioned on Allen's frame in which thorax and abdomen are completely free. Defibrillator pads can also be applied to a prone patient and shocks can be delivered, though pad application can be challenging in a patient who is already draped and positioned for surgery [39]. Nonetheless, the surgeon can initiate prompt CPR in view of sterile surgical field (mainly in spine surgeries) and anesthesiologist can manage intravenous medications as well as other related tasks.

CPR has been successfully applied in lateral and sitting position [7]; however, there is a lack of standard guidelines related to management of patients in these positions. Cases of successful cardiopulmonary resuscitation in the lateral position have also been explored and reported in the neurosurgical patients [21, 53]. In one case of ventricular fibrillation arrest, two rescuers simultaneously applied the compressions to the sternum and the back, while the dura was closed, and preparations were made to turn the patient supine [55]. In this case, defibrillation could not be applied in the lateral position due to the covering of thorax by a body-fixation device. Defibrillation was performed once the patient was repositioned [53].

Our search yielded few cases of cardiac arrest occurring in a patient undergoing elective craniotomy in the sitting position [18, 30, 38, 55]. The sitting position may predispose to hemodynamic complications due to venous air embolism or tension pneumocephalus [62]. Surprisingly, these cases were not related to venous air embolism. These occurred due to nerve root irritation and the episodes of asystole were transient and self-resolving with removal of surgical stimulus [38].

Interestingly, Beltran et al. in their report highlighted that, sometimes, even turning the position to supine from the lateral position could not revive the patients as torrential bleeding from skull base or spine could not be controlled in supine positions. Therefore, turning to prone in such circumstances may be a better choice [54].

## 5. Skull Pin Fixation

In the situation of asystole or ventricular fibrillation, cardiopulmonary resuscitation (CPR) and defibrillation are the management of choice; however, these methods may have serious implications with skull pins in situ. First, neck position during CPR is of major concern. In spite of muscle relaxant, serious cervical spine injury could occur; however, use of muscle relaxant ensures the prevention of sudden bucking/gagging during recovery phase (as some of the cardiac emergencies are transient in nature) and may be considered. Furthermore, defibrillation may cause surgical site injury and cervical spine injury due to body jerks. It is also a commonly held belief that defibrillation could lead to burns at the site of skull pin fixation; however we did not find any reports of this complication during our literature search.

Specific management of skull pins was rarely mentioned in the reviewed reports. It is possible that the above injuries were avoided by removing skull pins, but unfortunately this information was not included in the reviewed cases. Providing there is no concern for cervical injury, cardiopulmonary resuscitation and DC shock should be undertaken immediately [56]. However, we propose that skull pins should first be removed in cases with unstable cervical injuries or in cases where body jerks may cause these injuries.

## 6. Management

The neurosurgical population is a distinct subgroup of patients in which ACLS protocol cannot always be applied directly during the episodes of cardiac arrest. It is frequently impractical to proceed immediately with ACLS algorithms, due to patient positioning and surgical exposure. Furthermore, it is frequently necessary to immediately address the underlying cause of arrest, and as such it is crucial to have a thorough understanding of underlying causes as well as their management.

In contrast with elective procedures, severe adverse cardiac events in emergency neurosurgical cases occur mainly due to raised ICP. Brain codes, analogous to the Code Blue in ACLS, have been developed for the prompt management of cardiac arrest associated with emergent neurosurgical cases. When elevated ICP or brain herniation occurs, the so-called emergency neurological life support (ENLS) should be initiated, which includes the stepwise management of these events [63]. However, our review indicates that cardiac arrest in elective neurosurgery is only rarely due to elevated ICP; therefore ENLS cannot be applied uniformly.

**Figure 1:**
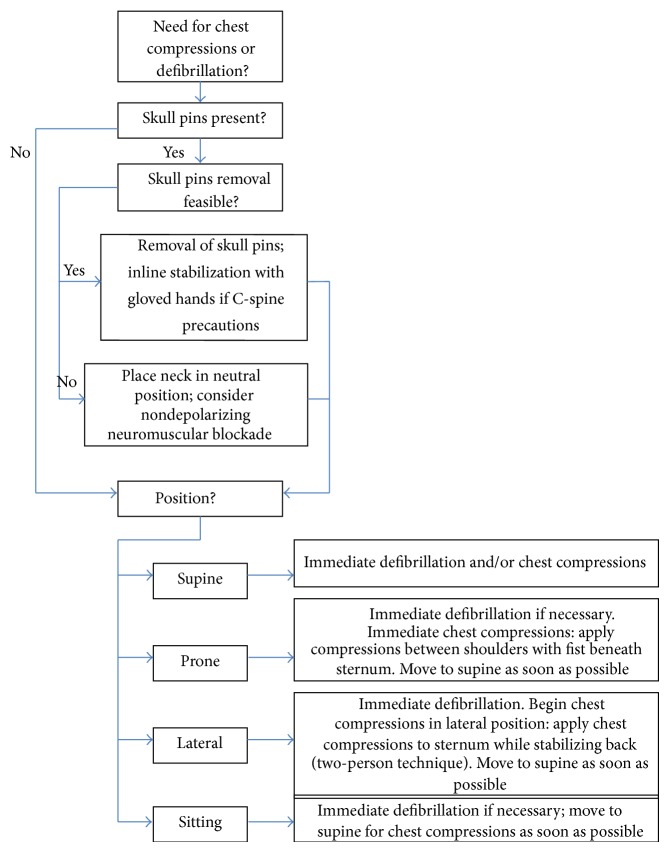
Algorithm for the management of cardiac arrest in neurosurgical patients with Mayfield fixation and nonsupine positions.

**Figure 2:**
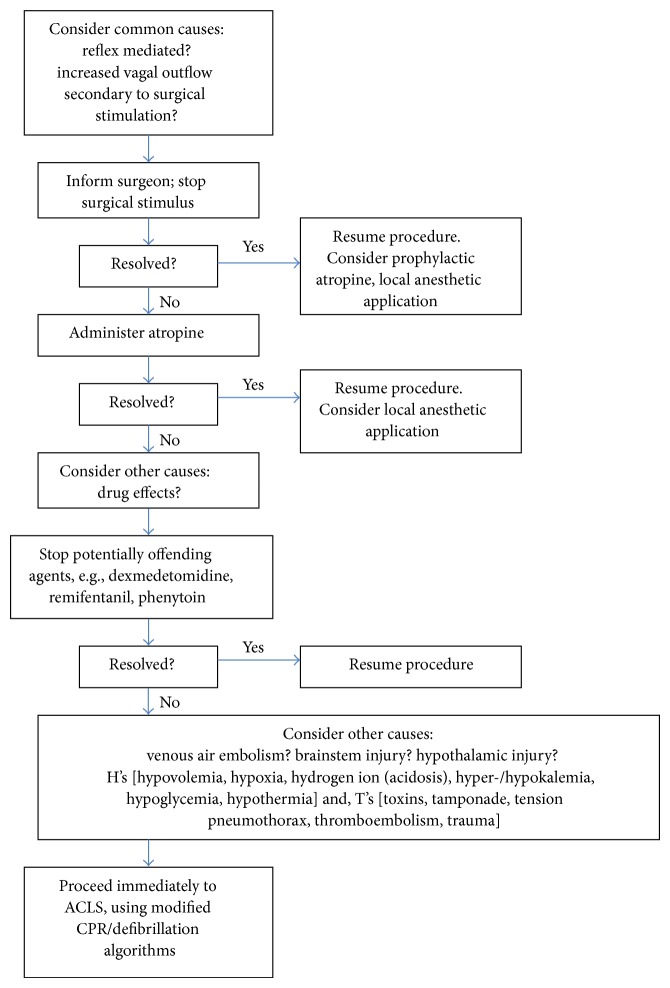
Management algorithm for bradycardia and asystole in neurosurgical procedures.

Below, we provide a summary of causes of cardiac arrest in elective neurosurgery, for both craniotomy and spine surgery. Based on this information, we propose a protocol to guide management of these catastrophic events (Figures 1 and 2).

### 6.1. Craniotomies

The common causes of sudden cardiac emergencies before the operative procedures include anesthetic induced, anaphylaxis, raised ICP due to aneurysm bleed, and stroke. On the other hand, neurogenic stimulation was described as the most common cause for cardiac arrest during surgical procedures. The commonly reported mechanisms of neurogenic stimulation were TCR, hypothalamic stimulation, brainstem stimulation, other cranial nerve stimulations, or stimulation of the limbic system, and venous air embolism. Of these mechanisms, TCR was the most frequently reported mechanism and was often aborted by stoppage of surgical stimuli; however, recurrent or persistent episodes of asystole should be managed with IV anticholinergic medication and adrenaline. Rarely these patients need CPR. On the other hand, drug-induced cardiac arrest was also an important cause of intraoperative cardiac arrest (both preprocedural and intraprocedural period), with a seemingly higher need for CPR and a trend towards worse outcomes, as compared to other causes.

### 6.2. Spine Surgeries

The common causes of cardiac emergencies during operative spine procedures include massive blood loss, nerve root mediated parasympathetic stimulation, anaphylaxis, autonomic dysreflexia, spinal shock, and venous air embolism. Besides treatment with anticholinergic and adrenaline, CPR is frequently performed in most of the cases. CPR as well as defibrillation is performed in nonsupine positions with success in majority of cases. interestingly, some of the patients are resuscitated successfully without changing the position as well. In the cases of venous air embolism, management should also include use of 100% oxygen, fluid resuscitation, vasopressors, and aspiration of air through an appropriately situated central venous line. In the event of a massive venous air embolism, CPR has been found to break the air locks that can produce severe cardiovascular collapse.

## 7. Limitations of Review

The results of this review are subject to multiple limitations. First, virtually all publications on this topic are either case reports or case studies. Large observational studies potentially could provide more insight with regard to incidence and management of cardiac arrest in elective neurosurgery, but such studies would be difficult to conduct due to the rarity of the event in question. Randomized studies would be ethically problematic and poorly feasible due to the rarity of intraoperative cardiac arrest. Secondly, the available studies on this topic are heterogeneous, and many reports did not contain information that we feel is important. For instance, particular management of skull pin fixation in the setting of CPR and defibrillation was not mentioned in any of the reviewed papers. Also, neurological outcome was not consistently reported in all studies. When cardiovascular resuscitation was successful and neurological outcome was not specifically mentioned, we assumed that neurological outcome was good, which may not be accurate.

## 8. Conclusion

There is very limited evidence with regard to causes and management of cardiac emergencies especially, intraoperative cardiac arrest in elective neurosurgical population. This paper serves as the comprehensive review of this topic and suggests that the etiology of these events in neurosurgery is often different than in other settings. Based on the available information, management of cardiac catastrophes in this population should be modified, and we propose a management algorithm as described. Perhaps the majority of anesthesiologists will never encounter cardiac arrest during elective neurosurgery; however one never knows when one will be faced with such a situation. A proper understanding of the mechanisms and management options related to these events would certainly assist in minimizing adverse outcomes. The role of simulation should be addressed in this context in the near future.

## Figures and Tables

**Table 1:** Cardiac emergencies in supratentorial surgery.

Author	Patient (*n*) (age/sex)	Procedure	Cardiac rhythm	Cause	Management	Outcome
Chowdhury and West [3]	50 y ♀	Awake craniotomy (frontal tumor)	Bradycardia, asystole (25 s)	TCR (scalp traction)	Release scalp traction; cessation propofol and remifentanil infusions	No neurological deficit
Prabhu et al. [4]	65 y ♂	Awake craniotomy (temporal tumor)	Bradycardia and asystole (8–10 s × 2 times)	TCR (dura cautery)	Cessation of cautery, atropine	No neurological deficit
Sinha et al. [5]	18 y ♂	Epilepsy surgery	Bradycardia (2 episodes), asystole (9 s)	↑Parasympathetic response 2° to amygdala resection; warm and cold saline irrigation	Atropine; cessation of stimulus	No neurological deficit
Sato et al. [6]	6/42 [18–34 y; ♀ = 3, ♂ = 2]	Epilepsy surgery	Sinus bradycardia (<1 min)	↑Parasympathetic tone 2° to limbic stimulation	Procedure interruption; atropine	No neurological deficit
Tyler et al. [7]	22 y ♀	Craniotomy (parietal tumor)	Asystole	Intracranial hematoma (dural closure)	Hematoma evacuation	No neurological deficit
Wasnick et al. [8]	70 ♂	Epidural suction drain	Severe bradycardia	Intracranial hypotension	Release of suction	No neurological deficit
Alfery et al. [9]	18 ♀	VP shunt	VT, VF	Intracranial hypotension	Lidocaine, CPR	No neurological deficit
Karamchandani et al. [10]	65 ♀	Subgaleal suction drain	Multiple severe bradycardia	Intracranial hypotension	Release of suction, atropine	No neurological deficit
Bhagat et al. [11]	2 patients [both 60 ♂]	Subgaleal suction drain	Severe bradycardia, Asystole	Intracranial hypotension	Release of suction, atropine	No neurological deficit

♀ = female gender; ♂ = male gender; TCR = trigeminocardiac reflex; no neurological deficit = postoperative deficit not mentioned.

**Table 2:** Cardiac emergencies in skull base surgery.

Author	Patient	Procedure	Cardiac rhythm	Cause	Management	Outcome
Cho et al. [12]	3 patients [28–42 y; ♀ = 2, ♂ = 1]	Transsphenoidal pituitary resection	Asystole (*n* = 2); bradycardia (*n* = 2)	TCR (cavernous sinus manipulation)	Cessation of surgical manipulation	No neurological deficit
Abou-Zeid et al. [13]	26 y ♀	Transsphenoidal pituitary resection	Asystole (30 s)	TCR (cavernous sinus manipulation)	Atropine	No neurological deficit
Seker et al. [14]	53 y ♂	Transsphenoidal pituitary resection	Asystole (20 s)	TCR (cavernous sinus manipulation)	Cessation of surgical manipulation; atropine	No neurological deficit
Meng et al. [15]	1/100 patients (skull base surgery) [56 ♀]	Sphenoid wing meningioma	Bradycardia	TCR	None	No neurological deficit
Reddy et al. [16]	40 y ♂	RF-ablation (trigeminal neuralgia)	Asystole and bradycardia (<1 min)	TCR	Atropine	No neurological deficit
Rath et al. [17]	50 y ♂	Transsphenoidal pituitary surgery	Asystole (10 s); pulseless bradycardia (30–40 s)	Hypothalamic injury	Atropine; CPR	CN 6 palsy, blindness
Stauber et al. [18]	34 y ♀	Pituitary adenoma resection	Asystole	TCR	Sternal punch, IV epinephrine	No neurological deficit
Ori et al. [19]	34 y ♂	Percutaneous thermocoagulation (petrous ganglion of Andersch)	Bradycardia, asystole (5 s)	GVR	Atropine, CPR	Hypalgesia of auditory canal

♀ = female gender; ♂ = male gender; TCR = trigeminocardiac reflex; GVR = glossopharyngeal-vagal reflex; CN = cranial nerve; CPR = cardiopulmonary resuscitation including chest compressions; no neurological deficits = postoperative deficits not mentioned.

**Table 3:** Cardiac emergencies in posterior fossa surgery.

Author	Patient	Procedure	Cardiac rhythm	Cause	Management	Outcome
Harada et al. [20]	69 y ♂	Craniotomy (CPA) meningioma)	ST elevation, VF	Coronary artery spasm secondary (vagal stimulation)	Procedure abandoned	No neurological deficits
Jaiswal et al. [21]	32 y ♂	Retromastoid suboccipital craniotomy	Bradycardia, asystole, VF, AF	TCR	Atropine, CPR, epinephrine, amiodarone	No neurological deficits
Usami et al. [22]	3 patients [36–52 y; ♀ = 2, ♂ = 1]	Temporal craniotomy (*n* = 1); retrosigmoid craniotomy (*n* = 2)	Asystole (*n* = 3)	TCR (*n* = 3), remifentanil (*n* = 3)	Atropine (*n* = 2); surgery interruption (*n* = 2)	No neurological deficits
Prabhakar et al. [23]	40 y ♀	Retromastoid suboccipital craniotomy	Asystole	TCR	Surgery interruption	Mild facial paresis
Schaller [24]	1/28	Microvascular trigeminal decompression	Asystole (33 s)	TCR	Surgery interruption	No neurological deficits
Sellery [25]	♀, age not specified	Chiari malformation	Asystole (20 s)	Brain stem manipulation	Atropine, ephedrine	No neurological deficits
Raman Sharma et al. [26]	60 y ♀	Retromastoid suboccipital craniotomy	Bradycardia (recurrent), asystole (40 s × 3 times)	Tumor excision (vagal stimulation)	Interruption surgery	Right vocal cord paralysis
Schaller et al. [27]	3/125 patients	CPA surgery	Asystole (<180 s)	TCR	Surgery interruption (*n* = 3), atropine (*n* = 3)	No neurological deficits
Loewenthal et al. [28]	53 ♀	Cerebellar meningioma	Bradycardia, asystole (3 min)	Coronary artery gas embolism; hypovolemia	CPR in prone position	No neurological deficits
Isabel et al. [29]	48 y ♀	Right retromastoid craniectomy,	Asystole (up to 45 s × many times)	GVR	Lidocaine, atropine; surgery interruption; transvenous pacing	No neurological deficits
Nagashima et al. [30]	74 y ♀	Suboccipital craniotomy (vagal rootlet section)	Asystole	Vagal stimulation	Atropine	Altered sensorium for 5 days

♀ = female gender; ♂ = male gender; CPA = cerebellopontine angle; TCR = trigeminocardiac reflex; GVR = glossopharyngeal-vagal reflex; no neurological deficits = postoperative deficits not mentioned.

**Table 4:** Cardiac emergencies in cerebrovascular surgery.

Author	Patient	Procedure	Cardiac rhythm	Cause	Management	Outcome
Kitabayashi et al. [31]	69 y ♀	Pterional craniotomy (Acom aneurysm)	Asystole (three episodes)	TCR (dural manipulation, remifentanil)	Atropine, release of stimulation	No neurological deficits
Spiriev et al. [32]	51 y ♀	Pcomm-ICA aneurysm clipping	Asystole	TCR with clip placement	Atropine, ephedrine	CN3 palsy
Kotake et al. [33]	54 y ♀	Aneurysm clipping	Bradycardia, complete AV block, VT/VF, PEA	Coronary vasospasm	Lidocaine, defibrillation, epinephrine	No neurological deficits
Faberowski and Gravenstein [34]	54 y ♂	Craniotomy (parietotemporal AVM)	VT	Myocardial ischemia	Withdrawal CVC, IV lidocaine, precordial thump, CPR (supine), defibrillation, epinephrine; surgery postponed.	No neurological deficits

♀ = female gender; ♂ = male gender; Acom = anterior communicating artery; TCR = trigeminocardiac reflex; AV block = atrioventricular block; VT = ventricular tachycardia; VF = ventricular fibrillation; PEA = pulseless electrical activity; CPR = cardiopulmonary resuscitation including chest compressions; no neurological deficits = postoperative deficits not mentioned.

**Table 5:** Cardiac emergencies in spine surgery.

Author	Patient	Procedure	Cardiac rhythm	Cause	Management	Outcome
Stauber et al. [18]	67 y ♀	Epidural steroid in cervical spine	PEA	Blockade of cardiac accelerator fibers	CPR, epinephrine	Mild cognitive dysfunction
Dooney [35]	43 y ♂	Lumbar discectomy	Asystole	Reflex vagal reaction due to dural traction	Atropine, adrenaline, CPR started in prone	No neurological deficits
Sharma et al. [36]	32 y ♂	Lumbar discectomy	Asystole	Intravascular ethanol injection	Atropine	No neurological deficits
Chen et al. [37]	75 y ♀	Lumbar vertebroplasty	Severe bradycardia	Fat embolism syndrome	CPR	Death
Hoell et al. [38]	60 y ♀	Cervical discectomy	Asystole (20 s)	Decreased sympathetic activity (anterior root irritation)	Interruption surgery	No neurological deficits
Brown et al. [39]	60 ♀	Thoracic spine decompression	Pulseless VT	VAE	Defibrillation	No neurological deficits
Lin et al. [40]	21 y ♀	T2-3 sympathectomy	VF	Sympathetic stimulation (stellate ganglion)	CPR	No neurological deficits
Raptis et al. [41]	24 cases	Lumbar discectomy	Hypotension, cardiac arrest	Vascular injury, hemorrhage	Laparotomy	Death in 7/24
Chow et al. [42]	22 y ♀	T2 sympathectomy	VF (<1 min), bradycardia, complete AV block, asystole (1 min)	↑Vagal tone	Atropine, epinephrine, CPR	No neurological deficits
Albin et al. [43]	2 patients [both 40 y ♂]	Lumbar laminectomy	Asystole (*n* = 1); PEA (*n* = 1)		CPR	Death
Ewah and Calder [44]	26 y ♀	L4-5 discectomy	PEA	Aortic laceration	CPR, ephedrine, epinephrine, crystalloid, colloid	Death
McCarthy et al. [45]	18 y ♀	Posterior spinal fusion	Hypotension, cardiac arrest	VAE	CPR (supine), open cardiac massage, vasopressors	No neurologic deficits; death 2° to multisystem failure at 2 weeks
Dumont et al. [46]	38 y ♂	Atlantoaxial arthrodesis	Asystole (minutes)	VAE	Surgical field irrigation, rapid skin closure, CPR (supine)	No neurological deficits

♀ = female gender; ♂ = male gender; VF = ventricular fibrillation; VAE = venous air embolism; CPR = cardiopulmonary resuscitation including chest compressions; no neurological deficits = postoperative deficits not mentioned.

**Table 6:** Drug-induced cardiac emergencies in neurosurgery.

Author	Patient	Procedure	Cardiac rhythm	Cause	Management	Outcome
Bharati et al. [47]	5 patients [51–76 y, ♂ = 5]	Lumbar laminectomy (*n* = 1); cervical discectomy (*n* = 2); craniotomy (*n* = 3)	VT (*n* = 1); asystole (*n* = 3); bradycardia (*n* = 1); PEA (*n* = 2)	Dexmedetomidine	CPR (*n* = 5), dopamine infusion (*n* = 3)	No neurological deficits
Baltaci et al. [48]	50 y ♂	Pterional craniotomy (ICA aneurysm)	Bradycardia, sinus arrest (60 min)	Locally applied papaverine	Atropine, CPR	Death
Braz et al. [49]	2/18 (cardiac arrest cases)					
	27 y ♀	Resection (vertebral column metastases)	Not specified	Asystole after IV metoprolol	CPR	Death
	37 y ♂	Cerebral abscess drainage	Not specified	Asystole after propofol	CPR	No neurological deficits
Miyahara et al. [50]	87 y ♂	Cervical laminectomy	VF (5 min)	Hyperkalemia	CPR	No neurological sequelae
Berry et al. [51]	49 y ♀	Craniotomy (aneurysm clipping)	Asystole (5 min)	Phenytoin overdose	CPR; epinephrine, calcium chloride, atropine	Drowsiness to postoperative day three
Lin et al. [52]	59 y ♀	Craniotomy (metastatic brain tumor)	Hypotension, bradycardia, complete AV blockade, asystole	Ephedrine with phenytoin infusion	Epinephrine	Good

♀ = female gender; ♂ = male gender; ICA = internal carotid artery; VT = ventricular tachycardia; VF = ventricular fibrillation; PEA = pulseless electrical activity; CPR = cardiopulmonary resuscitation including chest compressions; no neurological deficits = postoperative deficits not mentioned.

**Table 7:** Cardiac emergencies in different neurosurgical positions.

Author	Patient (age/sex)	Procedure	Cardiac rhythm	Change in position	Management
*Lateral, park bench *					
Takei et al. [53]	61 y ♂	Microvascular decompression	VT, VF	Yes	CPR lateral
Jaiswal et al. [21]	32 y ♂	Retromastoid suboccipital craniotomy	Bradycardia, asystole, VF, AF	No	Atropine, CPR, epinephrine, amiodarone
Usami et al. [22]	3 patients [36–52 y; ♀ = 2, ♂ = 1]	Temporal craniotomy (*n* = 1); retrosigmoid craniotomy (*n* = 2)	Asystole (*n* = 3), bradycardia (*n* = 2)	No	Atropine (*n* = 2); surgery interruption (*n* = 2)
Prabhakar et al. [23]	40 y ♀	Retromastoid suboccipital craniotomy	Asystole	No	Surgery interruption
Raman Sharma et al. [26]	60 y ♀	Retromastoid suboccipital craniotomy	Bradycardia (recurrent), asystole (40 s, three episodes)	No	Surgery interruption
Isabel et al. [29]	48 y ♀	Right retromastoid craniectomy, (glossopharyngeal neuralgia)	Asystole, multiple episodes (up to 45 s)	No	Lidocaine, atropine; surgery interruption; transvenous pacing
Beltran and Mashour [54]	2 patients [21 ♀, 69 ♀]	Craniotomy (neurofibroma) Lumbar corpectomy (metastasis)	Severe hypotension, PEA Severe hypotension, PEA	Yes Yes	Vasopressors, CPR, died Vasopressors, CPR, died
*Sitting *					
Stauber et al. [18]	67 y ♀	Epidural steroid in cervical spine	PEA	Yes	Epinephrine, CPR
Villeret et al. [55]	34 y ♀	Pituitary adenoma resection	Asystole	No	Sternal punch, IV epinephrine
Hoell et al. [38]	60 y ♀	Cervical discectomy	Asystole (20 s)	No	Interruption surgery
Nagashima et al. [30]	74 y ♀	Suboccipital craniotomy (vagal rootlet section)	Asystole	No	Atropine
*Prone *					
Dooney [35]	43 y ♂	Lumbar discectomy	Asystole	Yes	Atropine, adrenaline, CPR started in prone
Sharma et al. [36]	32 y ♂	Alcohol injection vertebral hemangioma	Asystole	No	Atropine
Miyahara et al. [50]	87 y ♂	Cervical laminectomy	VF (5 min)	Unknown	CPR, defibrillation, treatment of hyperkalemia
Chen et al. [37]	75 y ♀	Lumbar vertebroplasty	Severe bradycardia	Yes	CPR, died
Sellery [25]	♀, age not specified	Chiari malformation	Asystole (20 s)	No	Atropine, ephedrine
Brown et al. [39]	60 ♀	Thoracic spine decompression	Pulseless VT	No	Defibrillation
Faberowski and Gravenstein [34]	54 y ♀	Craniotomy (parietotemporal AVM)	VT/VF	Yes	Withdrawal CVC, lidocaine, precordial thump, CPR (supine), defibrillation, epinephrine; surgery postponed
Loewenthal et al. [28]	53 ♀	Cerebellar meningioma	Bradycardia, asystole (3 min)	No	CPR (prone)
Albin et al. [43]	2 patients [both 40 y ♂]	Lumbar laminectomy	Asystole in first and PEA in second patient	Yes	CPR, both died
McCarthy et al. [45]	18 y ♀	Posterior spinal fusion and instrumentation	Cardiac arrest	Yes	CPR (supine), open cardiac massage, vasopressors
Dumont et al. [46]	38 y ♂	Atlantoaxial arthrodesis	Asystole (minutes)	Yes	Surgical field irrigation, rapid skin closure, CPR (supine)
Miranda and Newton [56]	39 ♀	Debulking of tumor and internal fixation of third thoracic vertebra	VF	No	Defibrillation
